# Record-high positive refractive index change in bismuth germanate crystals through ultrafast laser enhanced polarizability

**DOI:** 10.1038/s41598-020-72234-w

**Published:** 2020-09-15

**Authors:** T. Toney Fernandez, Karen Privat, Michael J. Withford, Simon Gross

**Affiliations:** 1 MQ Photonics Research Centre, Department of Physics and Astronomy, Macquarie University, Sydney, NSW Australia; 2 Electron Microscope Unit, Mark Wainwright Analytical Centre, University of New South Wales, Sydney, NSW Australia

**Keywords:** Fluorescence spectroscopy, Raman spectroscopy, Glasses, Characterization and analytical techniques, Fluorescence spectroscopy, Raman spectroscopy, Laser material processing, Integrated optics

## Abstract

Unlike other crystals, the counter intuitive response of bismuth germanate crystals (${\text {Bi}}_4{\text {Ge}}_3{\text {O}}_{12}$, BGO) to form localized high refractive index contrast waveguides upon ultrafast laser irradiation is explained for the first time. While the waveguide formation is a result of a stoichiometric reorganization of germanium and oxygen, the origin of positive index stems from the formation of highly polarisable non-bridging oxygen complexes. Micro-reflectivity measurements revealed a record-high positive refractive index contrast of $4.25\times 10^{-2}$. The currently accepted view that index changes $>1\times 10^{-2}$ could be brought about only by engaging heavy metal elements is strongly challenged by this report. The combination of a nearly perfect step-index profile, record-high refractive index contrast, easily tunable waveguide dimensions, and the intrinsic high optical non-linearity, electro-optic activity and optical transparency up to $5.5\,\upmu {\text {m}}$ of BGO make these waveguides a highly attractive platform for compact 3D integrated optics.

## Introduction

Bismuth Germanate, ${\text {Bi}}_4{\text {Ge}}_3{\text {O}}_{12}$ (BGO) crystals are generally known as an efficient scintillation material for high energy radiation detection^1^. The versatility of the femtosecond laser direct-write technique to form localized high refractive index change ($\Delta {n}$) in BGO crystal has been proposed for applications such as two dimensional gamma ray spectrometers^2,3^, positron emission tomography^4^ and other optical waveguide devices^5,6^. The densely packed lattice of crystals makes it difficult to obtain an increase in refractive index upon ultrafast laser exposure^7,8^. As a result positive refractive index change has been reported only in a few selected crystals. Examples include a thermally stable type-I waveguides in ${{\text {Pr}}}^{3+}:{{\text {Y}}}_2{{\text {SiO}}}_5$ crystal^9^, colour-centres in LiF^10^, a transverse magnetic polarization guiding in potassium dihydrogen phosphate (KDP) crystal^11^, a change in the spontaneous polarization in ${{\text {LiNbO}}}_3$ that increases the extraordinary refractive index^7,12^, exploitation of the higher refractive index of amorphous silicon versus crystalline silicon^13^ and ultrafast laser-induced lattice defects in Nd:YCOB crystals^14^. But, those waveguides either suffer from a low refractive index change of the order of $10^{-3}$ or only guide a single polarization. This limitation is evident from the preferential choice of stress-induced or depressed cladding waveguides in crystalline media^7,8^. In contrast, BGO crystal was reported to produce waveguides of a smooth positive refractive index (type-I modifications^15^) in the thermal fabrication regime using megahertz pulse repetition rates^5^ as well as in the athermal regime at 1 kHz repetition rate^16^. The waveguiding properties, such as mode profiles and loss, has been reported elsewhere^5^.

This work explains for the first time the formation of the positive type-I index change of record high magnitude, which is unprecedented not only among crystals but for any dielectric medium. The combination of a record-high refractive index change, clean step-refractive index profile, easily customisable waveguide dimensions, wide wavelength transparency ranging from the UV to the mid-IR (0.3–5.5 $\upmu {\text {m}}$^17^), electro-optic activity and high optical non-linearity due to a bulk refractive index > 2.0^18^ could make BGO a powerful platform for ultrafast laser inscribed 3D photonic circuits.

**Figure 1:**
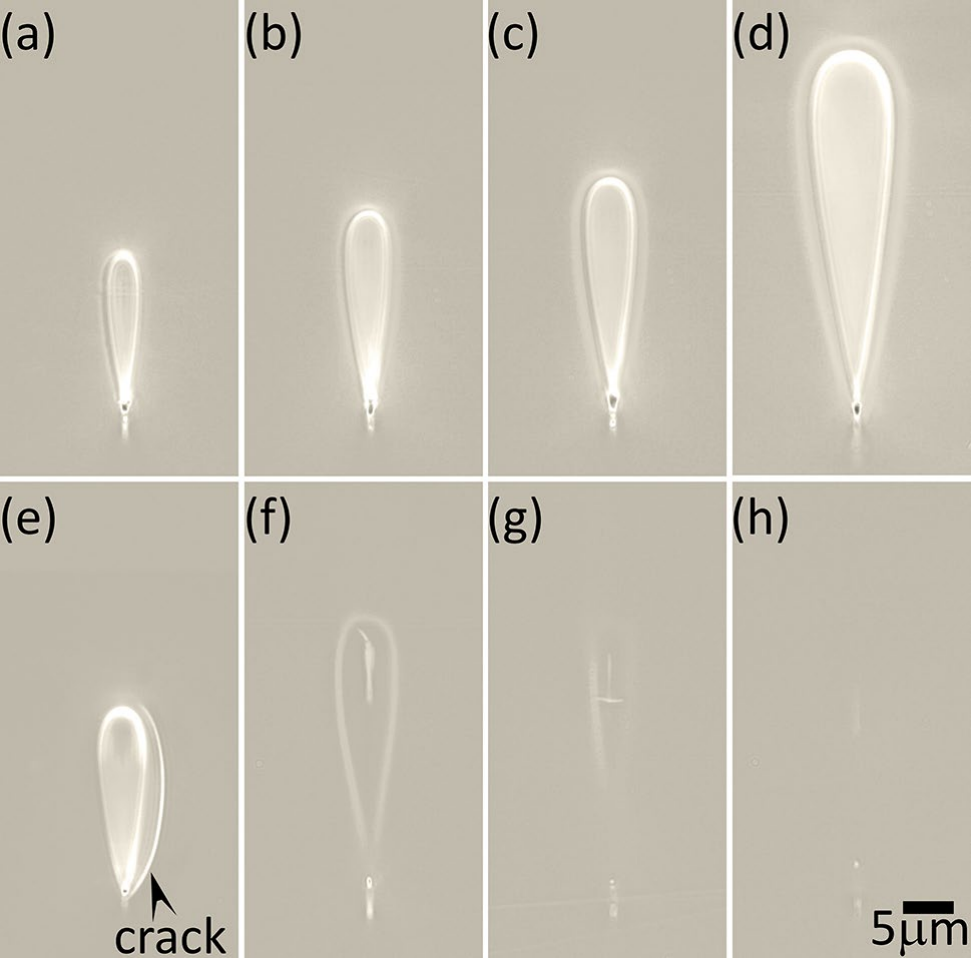
DIC images of waveguide end-faces written at 1,000 mm/min feed rate and energies of (**a**) 60 nJ, (**b**) 70 nJ, (**c**) 90 nJ, (**d**) 130 nJ and in the bottom row with an energy of 90 nJ at feed rates of (**e**) 4,000 mm/min, (**f**) 500 mm/min, (**g**) 200 mm/min and (**h**) 100 mm/min, respectively. The writing laser was incident from the top of the images.

## Results and discussion

Differential interference contrast (DIC) microscope images of the inscribed waveguides are shown in Fig. 1. Terminologies of “shell” which is the highly distinguishable peripheral halo and “core”, which is the region at the center will be used throughout the manuscript. The processing windows for type-I waveguides with smooth positive refractive index that are crack-free were found to be, 50–80 nJ for 2,000–4,000 mm/min and 50–130 nJ for 1,000 mm/min. Waveguides written with energies $\ge$ 90 nJ with 2,000–4,000 mm/min also exhibited a smooth positive refractive index change, but due to BGO’s high thermal expansion coefficient^18^ of $7\times 10^{-6}/^{\circ }{\text {C}}$, cracks formed along the boundaries between modified and unmodified material and were running down the waveguiding structures. The inverted tear drop structure of the waveguides can be attributed to a combination of spherical aberrations, Kerr-nonlinearity and an extended confocal parameter due to the high linear and nonlinear refractive index of BGO. For feed rates slower than 1,000 mm/min, an additional optically dense vertical filament-like structure became apparent within the core (Fig. 1f–h) . These filament structures likely resemble the shape of the laser induced plasma profile, as observed in phosphate glass^19^. This is supported by the observation of identical outer shell width irrespective of feed rates (Fig. 1c,f). Figure 1a–d shows waveguides written at 1,000 mm/min with energies ranging between 60 and 130 nJ. Figure 1e illustrates the crack formation at high feed rates (4,000 mm/min). All waveguides shown in Fig. 1e–g were written with 90 nJ energy. With decreasing feed rate the index contrast gradually vanishes due to slow quenching and self-annealing. The waveguides in Fig. 1c,f formed by identical pulse energy (90 nJ) but at feed rates of 1,000 and 500 mm/min, respectively, exhibit significant differences. Quench times of 30 and 60 ms were calculated^20^ from the time it takes to translate the sample by the average structure width of 5 um. Hence a 60 ms re-solidification window is too long to either produce a modification or provides enough time for the melt to re-attain its long range order.This suggests that in BGO the interplay between rapid thermal quenching and heat diffusion is critical for the formation of optical waveguides.

**Figure 2:**
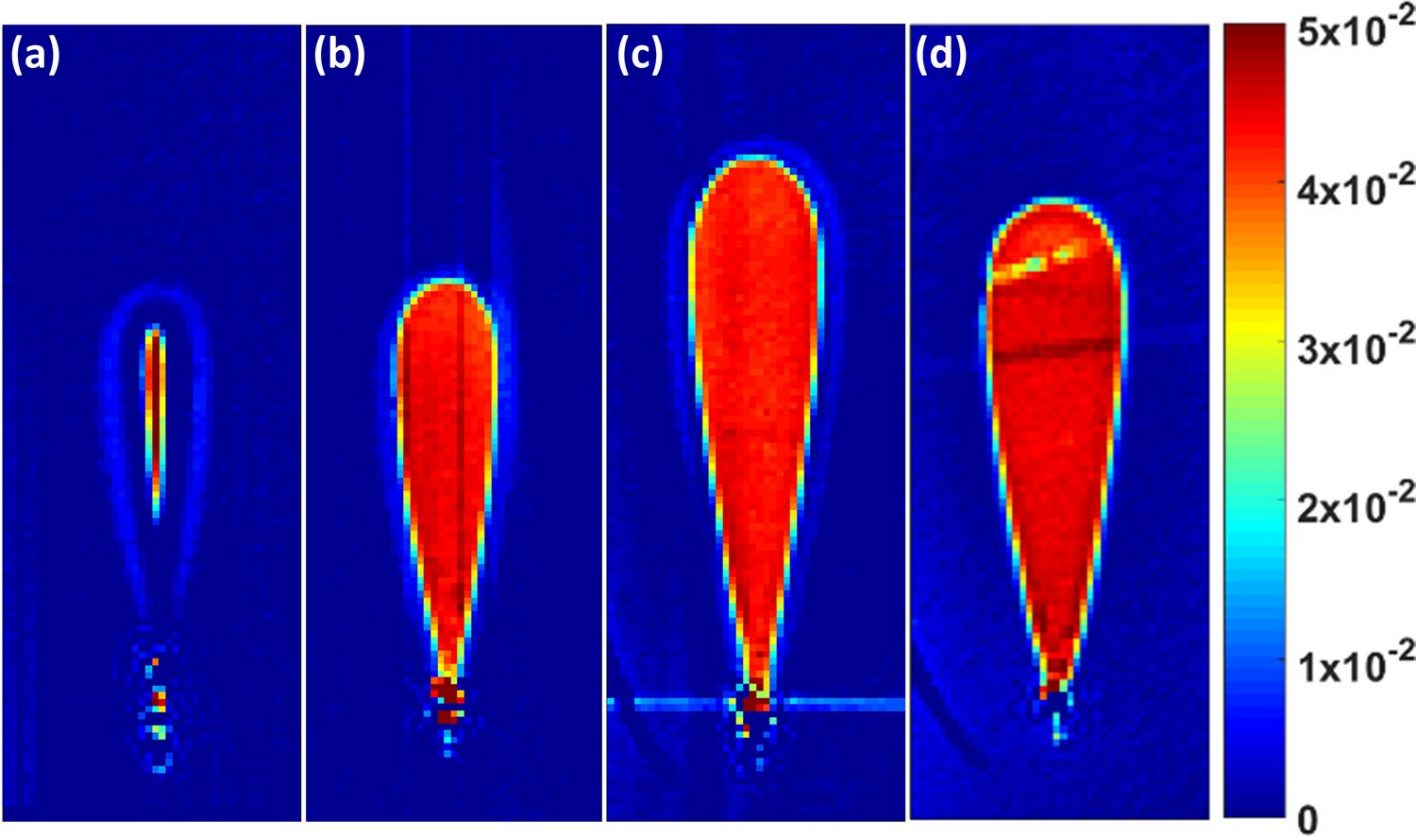
Refractive index profiles of waveguides written at (**a**) 90 nJ/500 mm/min, (**b**) 90 nJ/1,000 mm/min, (**c**) 120 nJ/1,000 mm/min and (**d**) 120 nJ/2,000 mm/min. Each map corresponds to an area of $15\times 40\,\upmu {\text {m}}$.

Micro-reflectivity ($\upmu$-PL) was used to measure the two-dimensional refractive index profiles (Fig. 2) at 833 nm with a spatial resolution of $\sim 0.25\,\upmu {\text {m}}$ (Olympus Plan N oil immersion microscope objective, $100\times$, 1.25 NA). The obtained 2-dimensional reflectance profile was converted into a refractive index profile using the Fresnel formula. The back surface was polished at $\sim 45^{\circ }$ to avoid retro reflections that affects the measurement. A record refractive index change value of $4.25\times 10^{-2}$ is observed for the waveguide inscribed at 1,000 mm/min. A value of $1.7\times 10^{-2}$ being the previous best was reported very recently using femtosecond-laser-induced electronic band-gap shift (FLIBGS) technique^21^. But the same was limited to a particular operating wavelength in visible. Also, unlike other previously reported peak index values in amorphous media, the index change in the current report is highly uniform throughout the core area and resemble a perfect step-index. Additionally, there is an absence of negative index zones, which are typically found for high-index waveguides in vitreous media^19,22^.

**Figure 3:**
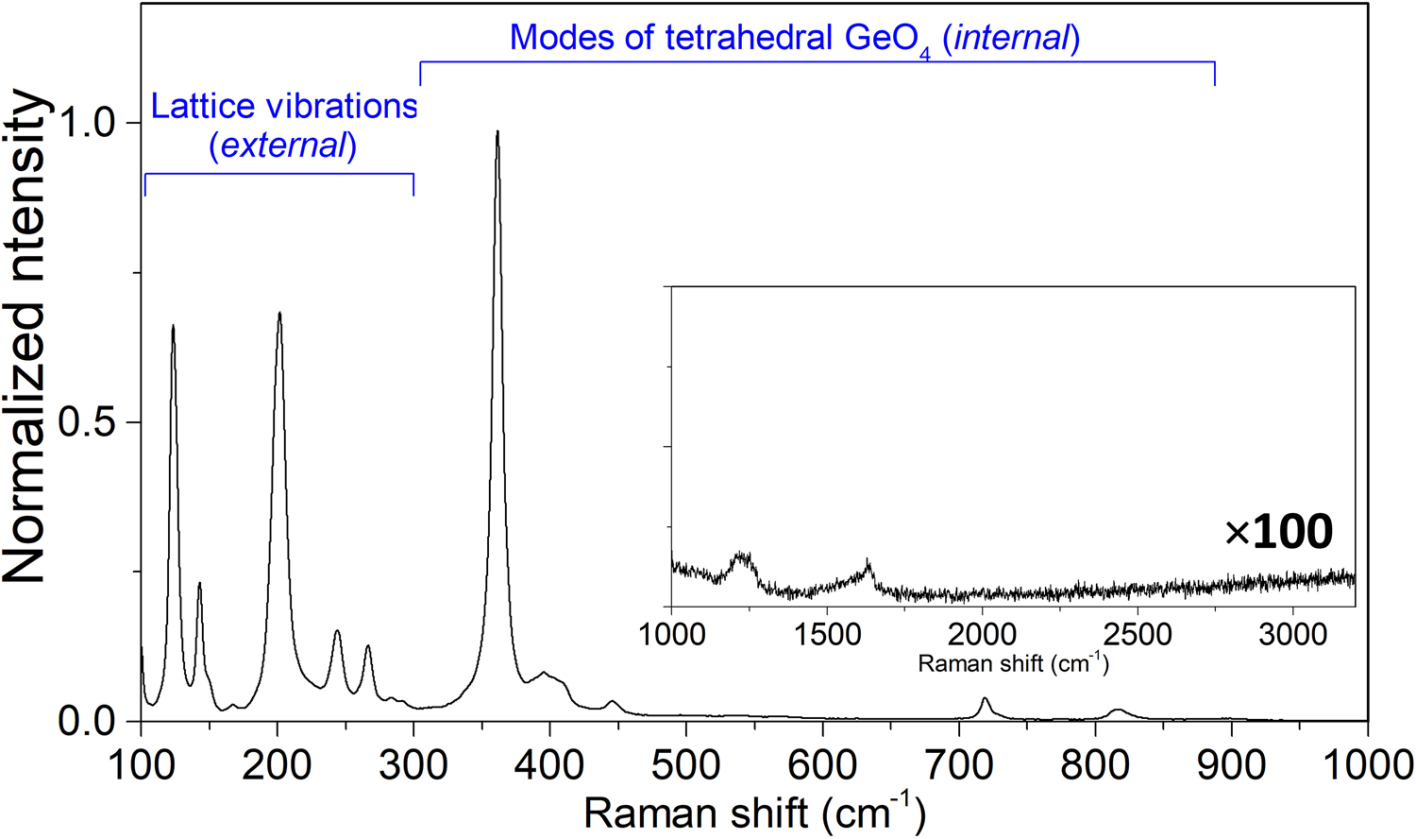
Raman spectrum of the BGO bulk crystal.

To reveal the structural dynamics of waveguide formation, micro-Raman spectroscopy was carried out using 633 nm excitation wavelength on a Renishaw inVia Raman microscope in confocal mode using a $100\times$ objective (spatial resolution $\sim 0.5\,\upmu {\text {m}}$). Figure 3 depicts the Raman spectrum of unmodified BGO exhibiting peaks between 65 and $820\,{\text {cm}}^{-1}$. The key features of BGO’s crystalline structure are (1) a distorted octahedral environment of oxygen around the ${\text {Bi}}^{3+}$ ions, (2) influence of heavier Bi–O atoms on the low Raman frequencies and (3) influence of lighter Ge–O bonds on high Raman frequencies^23,24^. The Raman spectrum of BGO is generally divided into two regions. The first region between $60{-}300{\,{\text {cm}}}^{-1}$, referred to as external vibrations, are the lattice vibrations corresponding to the motion of whole ${\text {GeO}}_4$ tetrahedral groups against the ${\text {Bi}}^{3+}$ ion sub-lattice. The second region between $360{-}820\,{\text {cm}}^{-1}$ corresponds to isolated ${\text {GeO}}_4$ tetrahera and is typically referred to as internal vibrations^23,24^. Since the vibrations are originating from a crystal, they are assigned to possible transverse optic (TO) or longitudinal optic (LO) phonons. The bulk crystal featured two additional Raman peaks at $1,210\,{\text {cm}}^{-1}$ and $1,627.5\,{\text {cm}}^{-1}$ (inset of Fig. 3), which have not been reported previously. Their high frequencies suggest that they are likely a combination mode from low frequency vibrations or vibrations of ${\text {H}}_{2}{\text {O}}$ that are trapped within the matrix. The elevated baseline at higher wavenumbers ($>2,000\,{\text {cm}}^{-1}$) is due to the luminescence tail caused by defects which will be discussed in detail later on.

**Figure 4:**
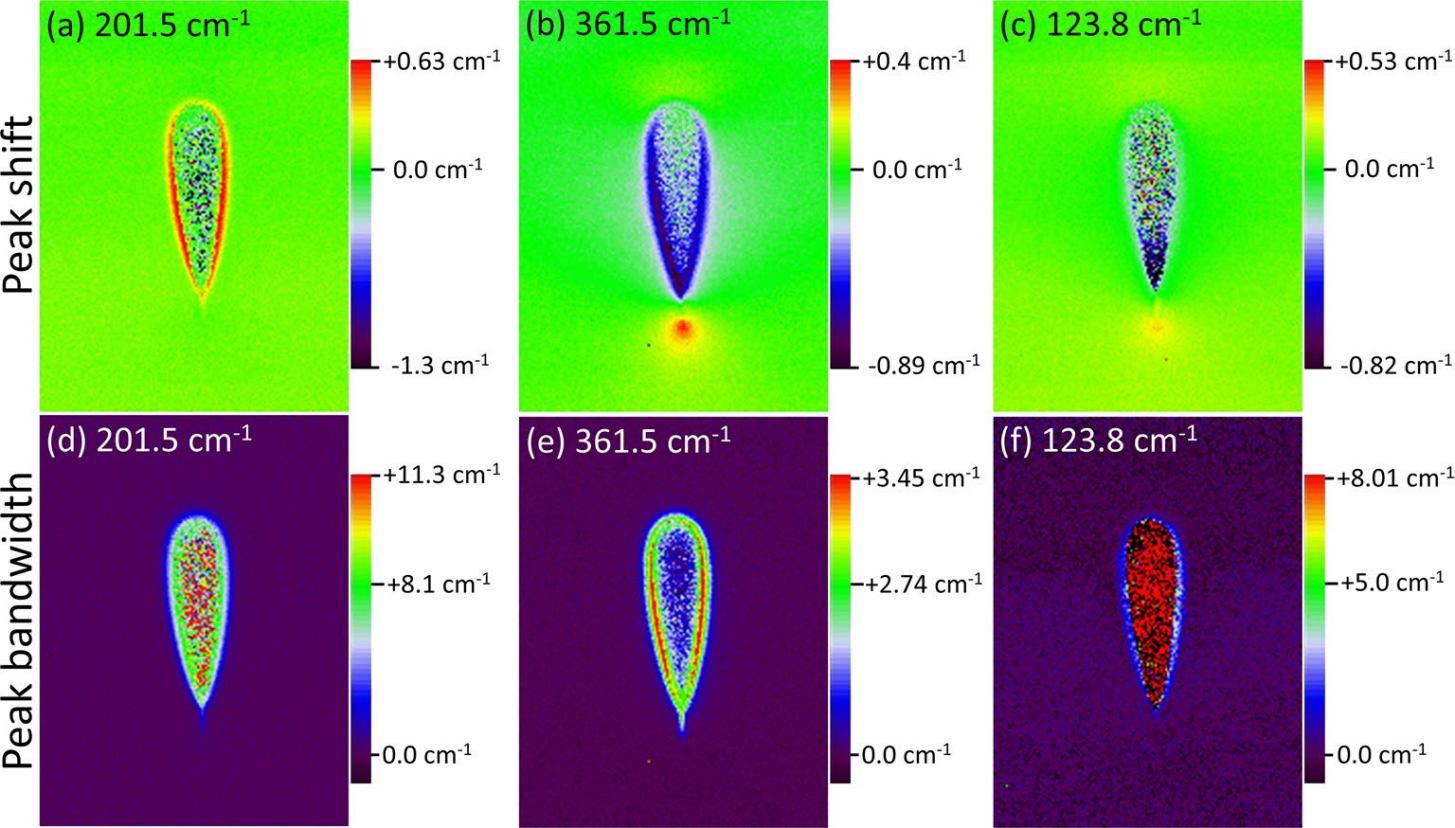
Raman maps of waveguide written at 90 nJ with 1,000 mm/min feed rate. First row shows shift in frequency and the bottom row the full-width at half maximum bandwidth (FWHM). Each map corresponds to an area of $30\times 40\,\upmu {\text {m}}$.

For Raman mapping a waveguide free of cracks, written at 90 nJ and with 1,000 mm/min, was chosen (Fig. 4). Figure 4a shows the spatial map of the peak at $201.5\,{\text {cm}}^{-1}$, which arises from lattice vibrations. While the peak frequency shift for lattice vibration ($60{-}300\,{\text {cm}}^{-1}$) was fairly low, the $201.5\,{\text {cm}}^{-1}$ band shows a distinct increase in frequency ($+0.3\,{\text {cm}}^{-1}$) in the shell (red pixels) and decrease in the core (dark blue pixels) relative to the pristine bulk. For the internal vibration, assigned to wavenumbers $>360\,{\text {cm}}^{-1}$, none of the peaks exhibit a peak shift, except for the $361.5\,{\text {cm}}^{-1}$ vibration (Fig. 4b). This band is assigned to the degenerate bending vibration of the isolated ${\text {GeO}}_4$ tetrahedron. Unlike for lattice vibrations, both core and shell regions exhibit a decrease in frequency shift, with the decrease more pronounced in the shell. The $361.5\,{\text {cm}}^{-1}$ band shows an increase in frequency at the bottom tip of the modification due to the mechanical stress typical for laser inscribed modifications in crystalline materials^7^. Additionally, the $361.5\,{\text {cm}}^{-1}$ band shows an increase in frequency at the bottom tip of the modification. This could be a result of mechanical stress, which is typical for laser inscribed modifications in crystalline materials^7^. Also, the lattice vibration at $123.8\,{\text {cm}}^{-1}$ exhibits a similar feature (Fig. 4c). The peaks at $361\,{\text {cm}}^{-1}$ and $123.8\,{\text {cm}}^{-1}$ arise from transverse optical phonons which have an additional magnetic field component in comparison to the longitudinal optical phonon vibration at $201.5\,{\text {cm}}^{-1}$. A detailed polarized Raman spectroscopy could reveal further information on the origin of this stress field.

By comparing the spatial morphology of the frequency shift of the $201.5\,{\text {cm}}^{-1}$ (lattice, low frequency vibrations) and $361.5\,{\text {cm}}^{-1}$ (internal, high frequency vibrations) peak, a pattern of inversion is evident. This is indicative of the migration of elements between the core and shell, since bismuth based vibrational units occupy the low frequency region ($<360\,{\text {cm}}^{-1}$) and germanium associated bands occupy the higher frequency regions ($>360\,{\text {cm}}^{-1}$). Mapping the distribution of FWHM (Fig. 4d–f) reveals an overall broadening of the vibrational bands in the laser modified regions with respect to the pristine bulk material. This indicates amorphisation within the structures. The lattice vibrations at $201.5\,{\text {cm}}^{-1}$ and $123.8\,{\text {cm}}^{-1}$ exhibit the strongest broadening in the core region. In contrast, the internal vibration at $361.5\,{\text {cm}}^{-1}$ only features a marginal bandwidth increase in both core and shell. Changes in bandwidths are more pronounced in comparison to peak shifts, which indicates that amorphisation is one of the key initiator for waveguide formation.

**Figure 5:**
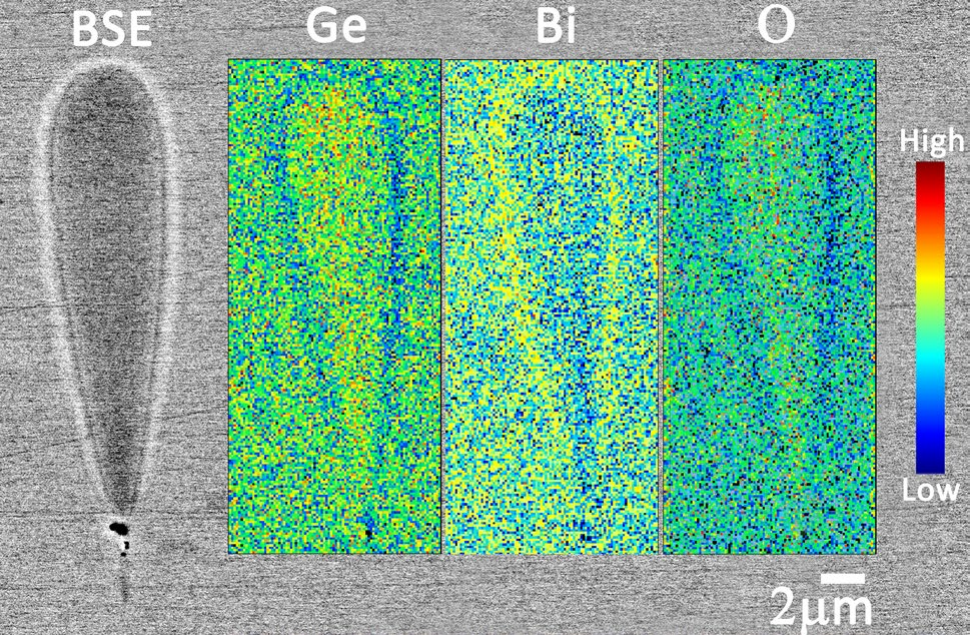
Backscattered electron image of the waveguide written at 90 nJ and 1,000 mm/min and its corresponding spatially resolved compositional map of Ge, Bi and O.

The field-emission backscattered electron (BSE) image, which gives contrast based on atomic number (Z-contrast) of the same 1,000 mm/min, 90 nJ waveguide reveals strong elemental variation across the waveguide’s cross-section (Fig. 5). The shell region (bright boundary) shows an increase in heavy elements, whereas relatively lighter elements are accumulated in the core (dark area). The BSE image rules out the possibility of refractive index increase through stress-induced localized density variation due to the rapid quench at high feed rates. If that was the case then the core would generate more backscattered electrons and a higher Z-contrast(psuedo) due to incident electron beam interacting with more nuclei (protons) of stress accumulated constituent atoms compared to the unirradiated zones^20^.The compositional maps (insets of Fig. 5) acquired using electron probe micro-analysis (EPMA) by utilising wavelength dispersive X-ray spectroscopy (WDS) clearly confirm elemental migration. A migration of the heavy bismuth (at. no. 83) to the shell and migration of the lighter germanium (at. no. 32) and oxygen (at. no. 8) to the core. The secondary electron image showed an entirely flat and featureless surface morphology except for a sub-micron void structure found at the focal point (black spot in Fig. 5 BSE image).

From the BSE image, the positive index in the shell can be explained by densification due to the migration of relatively heavy Bi (bright zones due to the at. no. contrast) but the positive index in the core could not be explained as it shows as a rarefied zone (darker zones)^20^. The increase of refractive index in the core due to the migration of elements that exhibit a higher electronic polarizabiliy ($\alpha _e$), as for instance observed in the case of chalcogenide glass^25^, can be ruled out since the discrepancy of polarizability between Ge ($0.137\times 10^{-24} {\text {cm}}^3$) and Bi($1.508\times 10^{-24} {\text {cm}}^3$) is more than one order of magnitude^26^. This indicates the possible formation of a large number of non-bridged oxygen in the core. Non-bridging oxygen possesses a significantly higher polarizability ($3.88\times 10^{-24} {\text {cm}}^3$) compared to bridged oxygen^27,28^. This is underpinned by the migration of oxygen towards the core observed using EPMA (Fig. 5, outmost right inset).

To investigate the presence and identify the type of non-bridged oxygen, confocal micro-photoluminescence ($\upmu {\text {PL}}$) was carried out using excitation lasers between 442 and 1,064 nm. Due to the wealth of publications based on Si compared to relatively few on Ge, the discussion here is based on Si as it is isochemical to Ge. No PL relating to interstitial ${\text {O}}_2$, ${\text {O}}_3$^29^, oxygen deficiency related centers (ODC)^30^ or Ge based non-bridging oxygen ($\equiv \text {Ge}{-}\text{O}^-$)^31^ was detected. The excess availability of oxygen in the core due to migration can induce the formation of non-bridging oxygen hole centers (NBOHCs) and/or interstitial molecular oxygen, which are generally found and reported in oxygen excess silica. From the experimental observation, no Raman vibrations or PL of interstitial ${\text {O}}_2$ (${\text {O}}+{\text {O}}\rightarrow {\text {O}}_2$, $\lambda _{ex} = 1{,}064\text { nm}$, $\lambda _{em} = 1{,}275\text { nm}$, ${\text {FWHM}} = \sim 12\text { nm}$) molecules were found and the luminescence of O$_3$ are usually in the UV region (${\text {O}}_2+{\text {O}}\rightarrow {\text {O}}_3$, $\lambda _{em}$ = 258 nm), which is below the UV absorption edge of BGO crystals^29^. It is possible that ${\text {O}}_{2}$ and ${\text {O}}_{3}$ are formed, but their existence might be a transient phase before they are converted to other defects. PL due to oxygen deficiency related centers (ODC)^30^ was absent or could not be observed with any of the excitation wavelengths. Since the core is enriched by the influx of oxygen, PL from the oxygen excess defects^30^ is expected, such as Ge based non-bridging oxygen ($\equiv \text {Ge-O}^-$, $\lambda _{ex} = 590{-}656\text { nm}$, $\lambda _{em} = 666\text { nm}, {\text {FWHM}} = \sim 58\text { nm}$)^31^ and/or peroxy linkage or peroxy radical center based defects.

**Figure 6:**
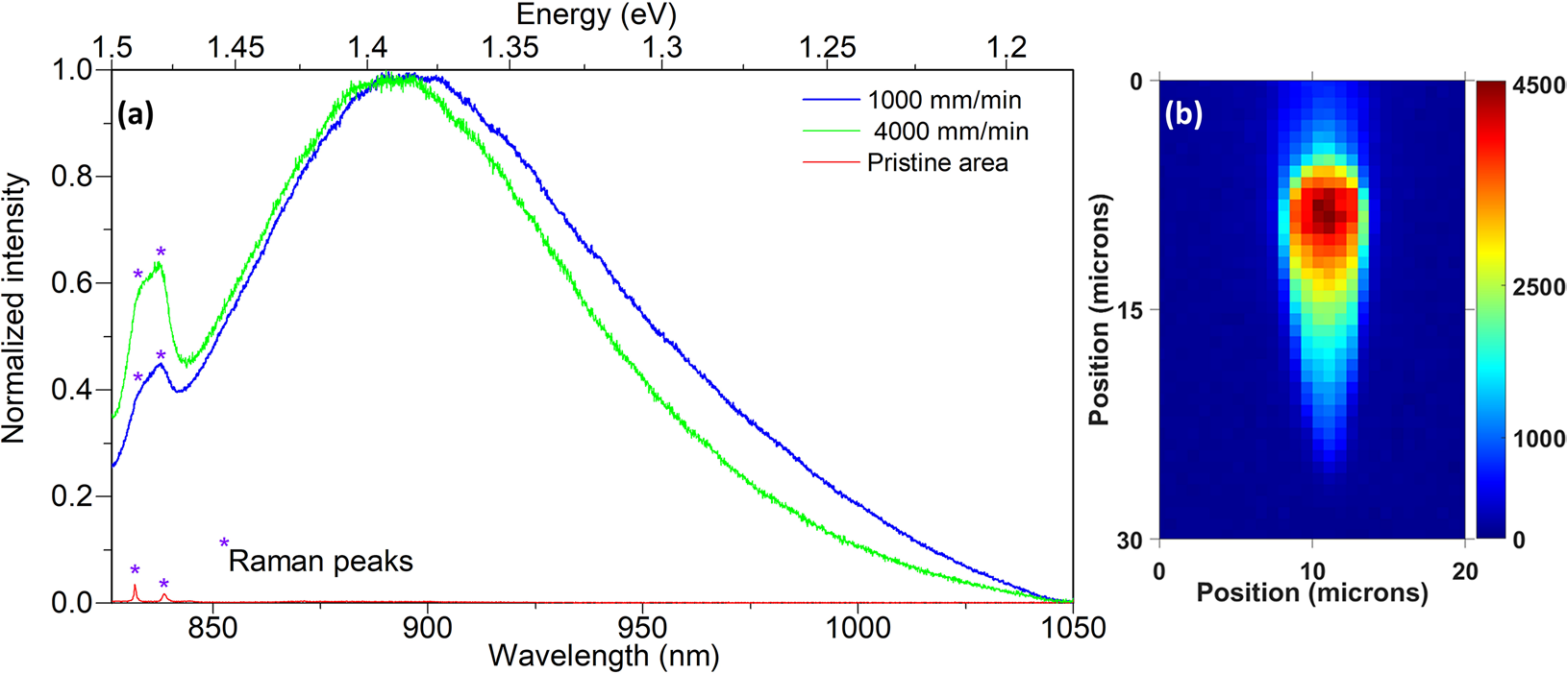
(**a**) PL spectrum from the core of waveguides written at 1,000 and 4,000 mm/min and from a pristine area using 785 nm excitation. (**b**) The 2-D intensity map of the PL across the waveguide written at 1,000 mm/min with 90 nJ of energy.

Of all the excitation wavelengths, only 785 and 830 nm laser wavelength gave a PL spectra centered between 890 and 900 nm for different waveguides, as shown in Fig. 6a. The PL was observed only from the core of the waveguide for feed rates between 1,000 and 4,000 mm/min. The bulk crystal did not show any PL (plotted in red in Fig. 6a). From the broad peak it can be understood that the PL arises from amorphous sites and no crystalline stark splitting is observed. The previous reports tentatively attributed the broad PL band centered at 867 nm (1.43 eV) to the presence of a higher energy lying (326 nm/3.8 eV for silica) absorption band^32^. This UV absorption band was in turn attributed to the presence of peroxy radicals or otherwise known as superoxide radicals ($$\equiv $$Si–O–O$$^*$$, $$^*$$ denoting an unpaired electron), which are formed when surplus oxygen molecules react with network vacancies ($$\equiv $$Si–Si$$\equiv $$ + O$$_2$$$$\rightarrow $$$$\equiv $$Si–O–O$$^*$$). In the present work, this mechanism is highly probable as transient vacancies could be formed due to the outflow of bismuth with such vacancies becoming available to the incoming oxygen to form peroxide radicals. The shift of PL towards a longer wavelength (lower energy) is expected due to the heavier germanium atoms ( $$\equiv $$Ge–Ge$$\equiv $$ + O$$_2$$$$\rightarrow $$$$\equiv $$Ge–O–O$$^*$$) when compared to their silicon counterparts^31^. The shift of approximately 0.052 eV in our case is very close to the previously reported energy shift of the relatively higher energy 1.9 eV PL band (non-bridging oxygen hole centers) in pure SiO$$_2$$ glass towards 1.86 eV in pure GeO$$_2$$ glass^31^. Figure 6b shows the spatial map of PL intensity for the 90 nJ and 1,000 mm/min waveguide. It indicates a higher density of peroxy linkages at the top of the core overlapping with the location of the vertical filament-like structure observed in Fig. 1f for waveguides written at lower feed rates. This suggests that the formation and distribution of such defects tend to follow a thermal gradient profile which is effectively the in-situ plasma profile as reported in phosphate glass waveguides (figures 4 & 8 in^19^). Hence shaping the plasma profile within the matrix should provide a direct tunability on the waveguide morphology, a key factor in waveguide design. Using the same excitation wavelengths (785 and 830 nm), the peroxy radical PL was absent for feed rates slower than 500 mm/min. This is also reflected in the absence of a positive index zone at the core (Fig. 1d).

**Figure 7:**
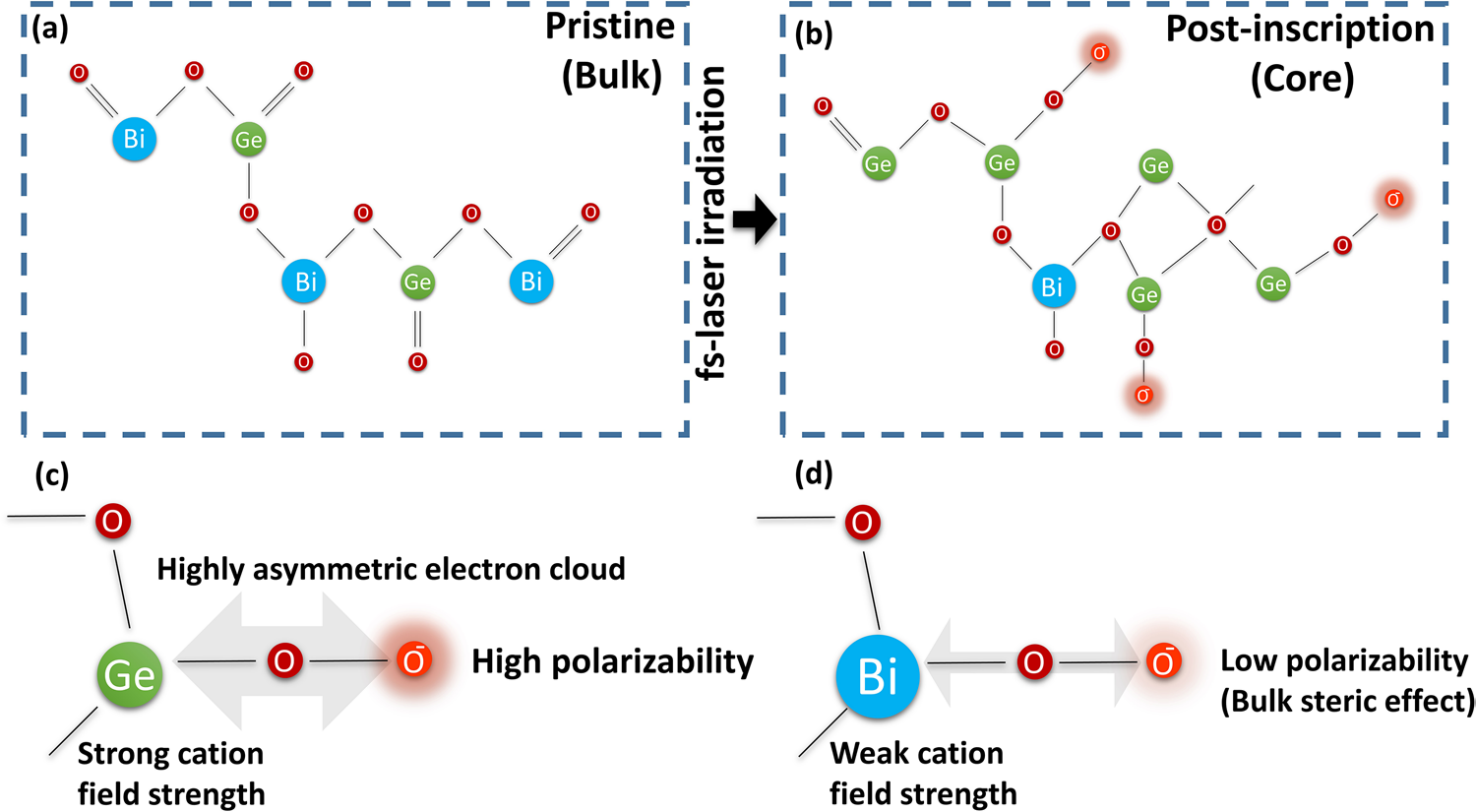
(**a**) The chemical structure of the pristine BGO crystal showing its long range order. (**b**) Post-inscription structure of the waveguide core that illustrates amorphisation and inward migration of Ge & O to replace Bi. (**c**) High polarizability induced due to strong asymmetry in the electron cloud caused by the strong cation field of Ge. (**d**) Steric hinderance effect due to the presence of Bi instead of Ge within the same chain.

Using the above evidence the waveguide formation can be explained as follows: As soon as the excitation/heat source is spatially displaced (at feed rates > 500 mm/min) and thus initiating fast quenching, oxygen migrates to the core, forming large numbers of superoxide radicals. This must occur within the re-solidification window. As a crystal with long range order (Fig. 7a), the bond breakage and formation of non-bridging oxygens can preferentially attract divalent cations to fulfill a cross link between two entirely different chains terminated with NBO. Germanium, as the divalent cation among the two glass formers, therefore undertakes paired migration towards the core with oxygen (Fig. 7b). However from the PL results it is clear that Ge migration is insufficient for a complete cross linking of NBOs (${\cdots }{\text {O}}^{-}\leftrightarrow {\text {Ge}}^{2+}{\leftrightarrow }{\text {O}}^-{\cdots }$). The magnitude of polarizability induced by the non-bridging oxygen depends on the linking cation field strength. The linked cation Germanium possesses a small ionic radius and a strong field strength, causing strong electron cloud asymmetry (Fig. 7c) that induces a high polarizability on the terminal NBOs which are present within its chain. Bismuth on the other hand suffers from a bulk steric effect due to its larger ionic radius and thus can only induce weak polarizability on terminal NBOs (Fig. 7d)^33^. This explains how the paired migration of Germanium and oxygen result in an increase of refractive index to such unprecedented high levels.

Utilizing polarizability of oxygen in glass making is a well known technique to obtain high refraction without engaging heavy elements. Also, a possibility of producing positive index waveguides in diamond by tuning its polarizability through ion beam damage was proposed recently^34^. But this report is the first instance where such a technique is fully exploited to produce and tune type-I waveguides, thereby demonstrating its superiority over other techniques^22^. Hitherto it was generally accepted that tunable index changes of $10^{-2}$ could be brought about only by engaging heavy metal elements within waveguiding structure^22^. While all other factors leading to density decrease (outflow of ${\text {Bi}}^{3+}$ and phase change from cyrstalline to amorphous) and molar refraction (outflow of ${\text {Bi}}^{3+}$) effectively work to lower the index, the observed increase in refractive index of $4.25\times 10^{-2}$ ($\approx 6.4\%$ increase from its base crystal refractive index) proves the importance of polarizability linked to anions. These observations provide more insights towards the future design of super-high refractive index waveguides in dielectric media induced by ultrafast lasers.

## Conclusion

To conclude, type-I waveguide formation by femtosecond laser direct-writing in a BGO is explained for the first time. Raman spectra demonstrated inverted behaviour of peak shift between external and internal vibrations, indicating migration of elements between the core and cladding of the inscribed structure. The large peak bandwidth increase for all the Raman bands indicated significant amorphisation within the structure. BSE and WDS confirmed the migration of bismuth to the peripheral shell and germanium and oxygen to the core. This revealed that the positive index core, with a record value of $4.25\times 10^{-2}$, is not formed due to densification or change in molar volume. A $\upmu {\text {PL}}$ spectroscopy from the waveguiding region confirmed the formation of large numbers of non-bridging oxygen associated to the peroxy radical. Non-bridged oxygen has a high polarizability compared to bridged oxygen causing the significant increase in refractive index.

## Methods

The waveguides were fabricated in commercially acquired BGO crystals (${\text {n}}_{D} = 2.109$^18^) using an extended cavity Ti:Sapphire chirped pulse oscillator (CPO), operating at a 5.1 MHz repetition rate, emitting sub-50 fs laser pulses at a center wavelength of 800 nm (FEMTOSOURCE scientific XL 500, Femtolasers GmbH). The circularly polarized laser beam was focused to a depth of $170\,\upmu {\text {m}}$ below the sample surface by a $100\times 1.25{\text {NA}}$ oil immersion objective (refractive of the immersion oil used was ${\text {n}}_D = 1.518$). To fabricate the waveguides, pulse energy was varied between 50 and 250 nJ and the feed rates between 10–4,000 mm/min. The micro-reflectivity technique was used to quantitatively measure the refractive index profile of the waveguides. For this purpose, the back surface of the BGO sample was polished at a $45^{\circ }$ angle to avoid back reflections from the waveguide end. The light from a single-mode fibre-coupled superluminescent diode ($\lambda =833\text { nm}$, FWHM = 20 nm) was focused by an Olympus $100\times$ oil immersion microscope objective (Plan N, NA 1.25) to achieve a spatial resolution/spot size of $\approx 0.3\,\upmu {\text {m}}$. The recorded 2-dimensional reflectance profile was converted into a refractive index profile using the Fresnel formula. Micro-Raman spectroscopy was carried out using 633 nm excitation wavelength on a Renishaw inVia Raman microscope in confocal mode using a $100\times$ objective (spatial resolution $\sim 0.5\,\upmu {\text {m}}$). The obtained spectra were deconvolved by pseudo-Voigt curve fitting to identify the Raman bands. A mapping of $30\times 40\,\upmu {\text {m}}$ area with waveguide as the center was done to understand the material response to ultrafast laser pulses. Micro-photoluminescence measurements were also conducted in confocal mode to maximize the resolution and restrict the light excitation and luminescence collection exclusively from the area of interest. A 100$\times$ objective with a spatial resolution of $\sim 0.5\,\upmu {\text {m}}$ was used. To reveal the elemental migration an electron probe micro analysis (EPMA) based on wavelength dispersive spectroscopy (WDS) was used. Scanning electron microscope imaging and X-ray intensity mapping of constituent elements were carried out on a JEOL JXA-8500F field-emission EPMA.

## Data Availability

The datasets generated and analyzed during the current study are available from the corresponding author on reasonable request.
